# Bacterial Mixology: Combining Pharmacodynamic Models to Predict In Vitro Competition of MCR-1-Harboring *E. coli*

**DOI:** 10.3390/antibiotics11010034

**Published:** 2021-12-28

**Authors:** Nicholas M. Smith, Arthur Chan, Thomas D. Nguyen, Jacob T. Dumbleton

**Affiliations:** 1 New York State Center of Excellence in Life Sciences and Bioinformatics, Buffalo, NY 14203, USA; tn34@buffalo.edu (T.D.N.); jacobdum@buffalo.edu (J.T.D.); 2 VA Medical Center, Buffalo, NY 14215, USA; Arthur.Chan2@va.gov

**Keywords:** *mcr*, antimicrobial resistance, Gram-negative bacteria, polymyxin resistance, *Escherichia coli*

## Abstract

The emergence of mobile colistin resistance (*mcr*)-mediated polymyxin resistance has resulted in a significant detriment to the utility of the polymyxins in the clinical setting. Though the risk for horizontal transfer of an *mcr*-containing plasmid is a major component of the transmissibility, selection of polymyxin resistant subpopulations is still a major risk factor for developing polymyxin-resistant infections. Using static time-kills over 24 h (h), we performed competition studies by mixing known inocula of isogenic *Escherichia coli* strains (wildtype [WT] and *mcr-1*-harboring) and treating with a concentration array of polymyxin B. These results were then compared to a priori predictions of bacterial-killing effects by polymyxin B on a mixed population of *E. coli* cells using a previously published mechanism-based model. The data showed that both selective pressure between WT and *mcr-1*-harboring strains as well as underlying polymyxin B heteroresistance within each of the two strains contributed to bacterial regrowth despite treatment with high concentration polymyxin B. Moreover, the simulations showed that when *mcr-1*-harboring cells were 1% or 10% of the total population, regrowth by 24 h was still observed in ≥50% of the simulated subjects for both a 10^6^ and 10^8^ inoculum. These results indicate that at lower inoculums with a low proportion of *mcr-1*-harboring cells, selective pressure from a pharmacokinetic-optimized regimen of polymyxin B still results in regrowth and selection of polymyxin-resistant cells.

## 1. Introduction

The recent proliferation of mobile colistin resistance (*mcr*)-mediated resistance among *Enterobacterales* has caused great concern in the clinical community [1,2]. The *mcr* gene exhibits diverse dissemination and has been identified in humans, animals, food, and environmental samples on every continent [3]. Species carrying the *mcr* gene are resistant to last-resort antibiotics and, subsequently, have the potential to cause pandemics with untreatable infections [4]. Since its first discovery in 2015, the number of *mcr*-harboring isolates has been increasingly reported worldwide at a concerning rate [5,6]. Because the *mcr* gene can undergo horizontal transfer there is the possibility for rapid spread of this resistance mechanism between and within a species [2,7,8]. Historically, polymyxin resistance has been due to chromosomally-mediated pathways, which manifests as a significant degree of heteroresistance [9]. Heteroresistance presents itself as resistant subpopulations that are capable of outcompeting other bacteria after polymyxin selective pressure is applied. Continued use of polymyxin is followed by increased rates of resistance and spread of transferable resistance genes contributing to the emergence of pan-drug-resistance [10].

Our group previously published a mechanism-based model of polymyxin B pharmacodynamics against isogenic strains of wildtype (WT)/polymyxin-susceptible and *mcr-1*-harboring/polymyxin-resistant *Escherichia coli* [11]. A main finding of this previous study was the underlying heteroresistance still present in the *mcr-1*-harboring isolate, which implies that selection for polymyxin B resistance can occur as either within strain selection (i.e., a given strain has a number of subpopulations with a given expression difference that confers resistance) or between strain selection (i.e., the strains have an underlying genetic difference that confers resistance). Another previously published study provided evidence that *E. coli* isolates producing *mcr-1* and New Delhi metallo-beta-lactamase 1 (*bla*_NDM-1_) were completely eradicated by a triple combination of polymyxin B, aztreonam, and amikacin at varying bacterial densities in an in vivo hollow fiber infection model (HFIM) [12]. Even in the presence of heteroresistance, polymyxin B may still play a fundamental role in treatment combinations against *mcr*-harboring isolates.

With the spread of *mcr*-harboring bacteria among humans and livestock, it is currently unknown how quickly a small subpopulation of *mcr*-positive cells can overtake the *mcr*-negative cells and what the effects of changing polymyxin B concentration may be on this selection. Competitive time-kill studies have been previously used to explore the pharmacodynamics of drugs against a heterogeneous population of bacteria with differing resistance phenotypes and can be incredibly helpful in delineating the differences between *mcr*-positive and -negative isolates [12].

Therefore, the objective of this study was to compare a priori simulations based on the previously published model to newly generated data of bacterial counts from in vitro competition time-kill studies in order to elucidate the effects of within and between strain selection of polymyxin resistance [11]. This was accomplished by creating multiple in vitro study conditions that have varying proportions *mcr-1* (mcr1a)/wildtype (WT) at the beginning of the experiment (i.e., 0 h), then exposing individual time-kill arms within each group to an escalating concentration array of polymyxin B.

## 2. Results

**Figure 1:**
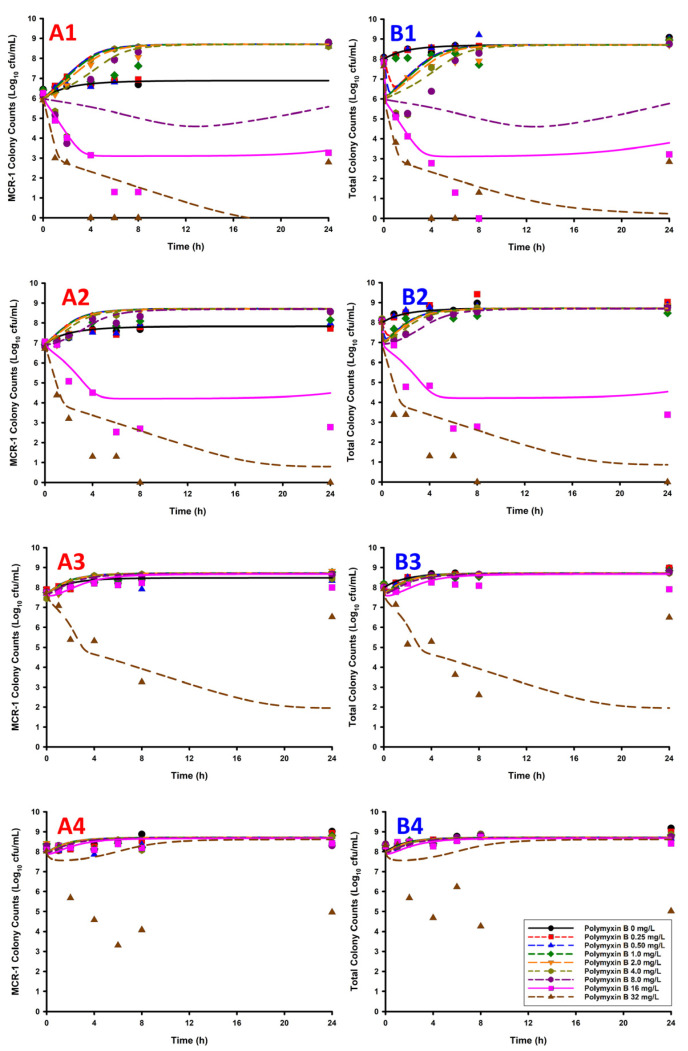
The listed graphs were generated by overlaying the observed bacterial counts from the observed static time-kill data (points) with the simulated bacterial count data (lines, i.e., predicted bacterial counts based on the previously published model) [11]. The counts of the mcr1a-only cells (**A1**–**A4**, mcr1a, only) and total population (**B1**–**B4**, mcr1a + WT) are represented by each column of panels. For the mcr1a-only panels/total population panels, the plots are listed as: 1% mcr1a + 99% WT (**A1**/**B1**), 10% mcr1a + 90% WT (**A2**/**B2**), 50% mcr1a + 50% WT (**A3**/**B3**), and 90% mcr1a + 10% WT (**A4**/**B4**).

The results of the a priori simulations of static polymyxin B concentrations against varying proportions of WT + mcr1a predicted a rapid initial killing followed by regrowth in cases where % WT > % mcr1a (Figure 1). In contrast, cases where % mcr1a ≥ % WT the simulations predicted an absence of bactericidal activity (defined as a ≤ 3 log_10_ cfu/mL reduction in bacterial counts) in all but one case. The one exception was when a polymyxin B concentration of 32 mg/L (roughly 21-fold higher than typical average steady-state concentrations in humans) was used against a 50% WT + 50% mcr1a, which predicted initial bactericidal activity.

**Figure 2:**
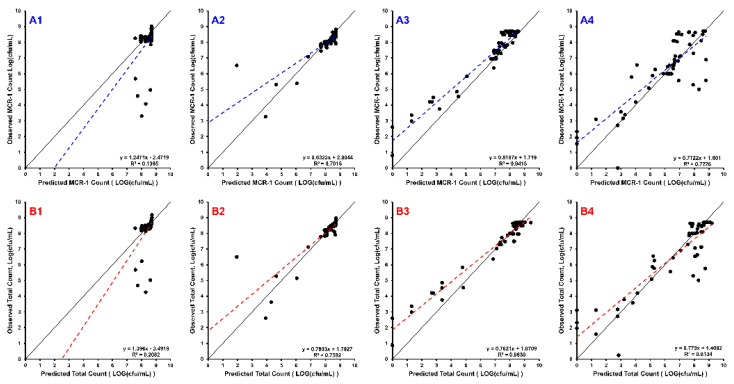
The line of identity (solid black) is overlain with the linear regression of the data (dashed line). Each row of sub-plots was divided so that **B1**–**B4** are the observed vs. predicted plots for the total bacterial population (WT + mcr1a), and **A1**–**A4** are the plots for mcr1a-only cells as determined by plating on kanamycin-containing plates. Each column of subplots represents the starting proportion of WT:mcr1a at the beginning of the experiment: 99% WT:1% mcr1a (**A1**,**B1**), 90% WT:10% mcr1a (**A2**,**B2**), 50% WT:50% mcr1a (**A3**,**B3**), and 10% WT:90% mcr1a (**A4**,**B4**).

The simulations were quantitatively assessed based on observed vs. predicted plots for both the total population (WT + mcr1a) and the *mcr-1* subpopulation (mcr1a-only), using linear regression to obtain slope and intercept, which had expected values of 1 and 0, respectively (Figure 2). All sets of observed vs. predicted plots were fit using a simple linear regression, which showed an adjusted R^2^ ≥ 0.70 and slope coefficient between 0.632 and 0.816 for cases where there were ≥50% WT cells in the culture. These metrics are not able to accurately quantify the 10% WT + 90% mcr1a culture conditions, as all concentrations result in growth of the system’s carrying capacity to ~10^9^ cfu/mL without significant killing effects, producing a cluster of points on the observed vs. predicted plot rather than a linear relationship.

## 3. Discussion

**Figure 3:**
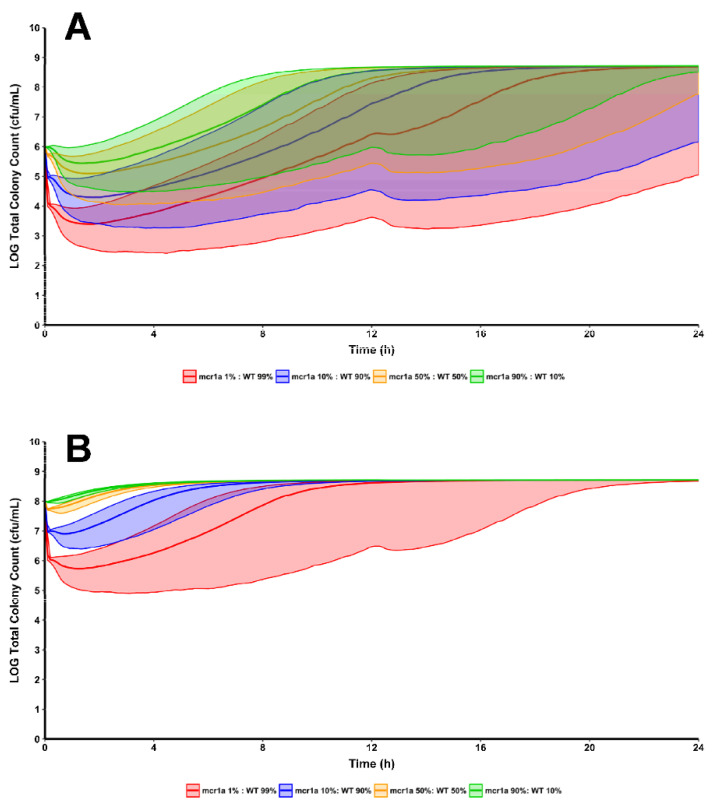
Using the same regimen structure implemented in the development of the mechanism-based model, simulations were conducted to see the effects of polymyxin B treatment against a bacterial population composed of varying proportions of WT and mcr1a cells. Simulations were conducted at both a 10^6^ cfu/mL (**A**) and 10^8^ cfu/mL (**B**) starting inoculum to explore the differences in regrowth pattern when there is an absolute increase in *mcr-1*-harboring cells, even if the proportion of cells remains constant.

Though bacterial killing effects produced by 32 mg/L of polymyxin B against 90% mcr1a + 10% WT were underpredicted, this does not greatly affect the translatability of the results. For reference, Polymyxin B 32 mg/L, is approximately 21-fold higher than the *f*C_ss,avg_ obtained from following guideline-recommended dosing and likely to be a supraphysiological concentration given the profound toxicities exhibited by the polymyxins. Supra-clinical and supra-physiological concentrations are important inclusions when prospectively validating a model as they permit exploration of a model’s ability to extrapolate beyond the original datasets. The observed versus predicted plots show a systematic bias in the model’s predictive performance; there was a clear ability to predict the trend of bacterial decline, nadir, and, if applicable, rebound. This reinforces the utility in utilizing multiple pharmacodynamic models in concert to generate rational hypotheses for situations where known resistant subpopulations are present. Future work could enhance the utility of this method by making use of many isolates in order to determine inter-strain pharmacodynamic variability, providing prediction intervals around simulations. Clinical trial simulations of 5000 patients per treatment group were conducted based on the pharmacokinetics of polymyxin B in critically ill patients, which were then used to drive the joint pharmacodynamic model [13]. Each treatment group received identical regimens of “front-loading” polymyxin B (3.33 mg/kg for one dose at 0 h, then 1.43 mg/kg Q12H starting at 12 h) but differed in the initial inoculum and the proportion of total population that were WT/mcr1a (Figure 3) [14]. Simulations of the population median and the 90% prediction intervals show that in all cases, bacterial regrowth was observed by 24 h for the 10^8^ cfu/mL starting inoculum for all proportions of WT:mcr1a and in >90% of simulated patients. However, for a lower 10^6^ cfu/mL starting inoculum and cases where the WT population was ≥90%, bactericidal activity was observed in a minority of patients (as indicated by the lower range of the prediction interval crossing below 10^3^ cfu/mL).

These data indicate that subpopulation selection between WT and mcr1a as well as within WT and within mcr1a populations is an important part of treatment failure. Previous studies found that polymyxin B exposure contributes to the subpopulation selection between WT and mcr1a resulting in the amplification of resistance showing polymyxin B dependency [15]. Higher concentrations of polymyxin B in this study exhibited initial bactericidal killing, supporting the “front-loading” approach of polymyxin B dosing in combination with other agents to combat resistance amplification [11].

Many clinicians and infection control personnel are wary of *mcr-1* given its ability to be horizontally transferred between species/strains. Though horizontal transfer is still a large concern at the population level, the newly generated in vitro and in silico data presented in this study show that even modest polymyxin B selective pressure (such as from inappropriate or sub-therapeutic therapy) is a more significant determinant in converting a minority population of bacteria harboring *mcr-1* into the majority population. Consequently, in the situation where a total population of bacteria has a low-level subpopulation of *mcr-1*-harboring cells, sub-therapeutic use of polymyxin B will result in rapid selection of the *mcr-1*-positive bacteria within the total population in addition to the selection of more polymyxin resistant bacteria within the *mcr-1*-positive subpopulation. These results are in agreement with previous reports illustrating the selection of resistant subpopulation even at nominal concentrations of antibiotic [16].

Additionally, we have shown via the simulation studies that clinically relevant dosing of polymyxin B against a population of *E. coli* that have a mixture of *mcr-1*-positive and -negative cells may be insufficient to produce eradication. This is of significance for individuals who may originate from or have traveled to *mcr* endemic areas, which have an increased likelihood of being colonized with *mcr*-harboring *Enterobacterales* [15]. Ultimately, understanding the confluence of intra- and inter-subpopulation selection is an incredibly important factor in determining the overall pharmacodynamic effect and development of resistance for polymyxin B.

The recently published guidelines for polymyxins have recommended that, for invasive infections due to carbapenem-resistant Enterobacteriaceae (CRE), a polymyxin should be used in combination with one or more additional susceptible agents [17]. Here, subpopulation-based synergy is defined as when two drugs exhibit synergy by providing coverage for different segments of the total bacterial population, which ensures that if one drug is ineffective on a given resistant subpopulation the other drug can still produce bacterial killing to eliminate that subpopulation. This highlights the importance of polymyxin B therapy in combination with other antimicrobial agents to combat multidrug resistant species. Combination therapy has shown the ability to treat pan-resistant isolates that are resilient antimicrobial monotherapy as bacterial killing effects occur through synergy between agents against phenotypically distinct subpopulations [18]. However, there is a need to optimize combination therapy, and this can be obtained through a data-driven, model-informed process to evaluate the effectiveness of combination drug therapies [19]. New polymyxin B dosing strategies have been explored that suggest certain antimicrobial combinations have the propensity to temporarily suppress polymyxin B resistance with active bacterial killing [18]. Additionally, the guidelines have further recommended the use of therapeutic monitoring for the reduction in toxicity and the prevention of resistance. The benefit of therapeutic monitoring for the prevention of resistance is underscored by the results of the competition time-kill and simulation studies, which show that careful dosing of polymyxin B in a combination is likely required to prevent *mcr*-harboring cells from supplanting wildtype cells and resistant subpopulations within each isolate from proliferating.

Altogether, these findings support the need for improved diagnostics to identify individuals who are candidates for polymyxin-therapy and who may be colonized with *mcr-1*-harboring organisms. Additionally, these findings support the use of a “front-loading” approach to dosing over standard dosing when polymyxin B is given and the likely need to use combination therapy when treating multidrug resistant organisms harboring *mcr*.

## 4. Materials and Methods

### 4.1. Bacterial Isolates, Antibiotics, and Media

All experiments were conducted using two *E. coli* strains: wildtype BW25113 (WT, MIC_PMB_ < 0.25 mg/L, MIC_Kanamycin_ = 1 mg/L) and BW25113-mcr1a (mcr1a, MIC_PMB_ = 8 mg/L, MIC_Kanamycin_ = 128 mg/L). MICs were determined via broth microdilution in at least duplicate. A target starting inoculum of 10^8^ cfu/mL was used for all experiments, where initial composition of the time-kills’ starting inoculum was adjusted to contain mcr1a and WT in ratios of (mcr1a:WT): 1%:99%, 10%:90%, 50%:50%, and 90%:10% [19]. Static time-kills were conducted using a 2-fold polymyxin B concentration array from 0.25 mg/L to 32 mg/L. Samples were collected at 0, 1, 2, 4, 6, 8, and 24 h and plated on both drug-free and kanamycin-containing (4 mg/L) Mueller-Hinton agar in order to differentiate between total and *mcr-1*-only populations, respectively.

### 4.2. Mechanism-Based Model

The previously published mechanism-based model of *mcr-1* in *E. coli* was used in simulations to predict responses in the static time-kills in order to prospectively validate the model for use in competition studies [11]. The logarithmic growth portion of the model was modified to account for both mcr1a and WT subpopulations. Resistant subpopulations whose initial bacterial concentration were determined to be less than 1 cfu per the assay volume of 20 mL were set to 0 cfu/mL as an initial condition. All simulations were conducted using Berkeley Madonna, with postprocessing and graphing being conducted in R (version 3.5.2) using the dplyr and ggplot2 packages.

### 4.3. Clinical Trial Simulation

To provide translational insights to the effects of clinically relevant dosing regimens and the effects of high inter-individual pharmacokinetic variability in critically ill patients, simulations were then performed to compare the consequences of the different types of subpopulation selection implemented on bacterial killing [13]. This approach was accomplished by utilizing the previously published population pharmacokinetics of polymyxin B in the critically ill [13]. The population pharmacokinetic model was combined with each of the two previously published pharmacodynamic models to create a new joint PK/PD model that would simulate hypothetical bacterial killing effects in response to clinically relevant dosing of polymyxin B (3.33 mg/kg ‘front-loading’, 1.43 mg/kg at 12 h). The median and 90th/10th prediction intervals were calculated by summarizing the Berkeley Madonna simulation output files in R.

## 5. Conclusions

The golden era of antibiotics is under serious threat with the emergence and proliferation of highly mobile resistance genes, such as *mcr*. It has been thought that selective pressure from prophylactic treatment of livestock resulted in rapid transfer and spread of the *mcr*-1 gene. Our results showed that even with lower proportions of *mcr*-1 harboring cells, selective pressure from polymyxin B resulted in re-growth and selection of polymyxin-resistant cells turning the minority population into the majority population. Polymyxin B use constitutes a major driving force for selection of the *mcr*-1 gene, which has limited its clinical utility globally. Because of this, the use of polymyxin B is heavily regulated in several countries to prevent further proliferation of *mcr*-mediated resistance. However, our findings show that the threat of *mcr*-mediated resistance may be combatted by utilizing the “front-loading” approach of polymyxin B within triple combination therapy. In treating future inevitable infections caused by *mcr*-harboring isolates, there is a need to optimize a combination regimen with polymyxin B that delivers the best clinical efficacy while minimizing toxicity. The use of in vitro infection models such as hollow-fiber systems can mimic human pharmacokinetics, which expands on static time-kill studies. Dynamic experiments utilizing hollow-fiber infection models provide insight in understanding emerging mechanisms of bacterial resistance such as the proliferation of the *mcr* gene. Understanding the effects of within and between strain selection of polymyxin resistance can provide clinical utility in optimizing treatments against *mcr*-mediated infections.

## Data Availability

Data and code are available from the authors upon reasonable request.
